# SAG2A protein from *Toxoplasma gondii* interacts with both innate and adaptive immune compartments of infected hosts

**DOI:** 10.1186/1756-3305-6-163

**Published:** 2013-06-05

**Authors:** Arlindo G Macêdo, Jair P Cunha, Thyago HS Cardoso, Murilo V Silva, Fernanda M Santiago, João S Silva, Carlos P Pirovani, Deise AO Silva, José R Mineo, Tiago WP Mineo

**Affiliations:** 1 Laboratório de Imunoparasitologia “Dr. Mário Endsfeldz Camargo”, Instituto de Ciências Biomédicas, Universidade Federal de Uberlândia, Av. Pará 1720 – Bloco 4C, Campus Umuarama, Uberlândia, Minas Gerais 38.400-902, Brazil; 2 Centro de Biotecnologia e Genética, Universidade Estadual de Santa Cruz, Rodovia Ilhéus/Itabuna km 16, Ilhéus, Bahia 45.662-900, Brazil; 3 Laboratório de Imunoparasitologia, Faculdade de Medicina de Ribeirão Preto, Universidade de São Paulo, Av. Bandeirantes, 3900, Ribeirão Preto, São Paulo 14.015-000, Brazil

**Keywords:** Toxoplasmosis, Protein modeling, SAG2A, Monoclonal antibodies, Recombinant protein

## Abstract

**Background:**

*Toxoplasma gondii* is an intracellular parasite that causes relevant clinical disease in humans and animals*.* Several studies have been performed in order to understand the interactions between proteins of the parasite and host cells. SAG2A is a 22 kDa protein that is mainly found in the surface of tachyzoites. In the present work, our aim was to correlate the predicted three-dimensional structure of this protein with the immune system of infected hosts.

**Methods:**

To accomplish our goals, we performed in silico analysis of the amino acid sequence of SAG2A, correlating the predictions with in vitro stimulation of antigen presenting cells and serological assays.

**Results:**

Structure modeling predicts that SAG2A protein possesses an unfolded C-terminal end, which varies its conformation within distinct strain types of *T. gondii*. This structure within the protein shelters a known B-cell immunodominant epitope, which presents low identity with its closest phyllogenetically related protein, an orthologue predicted in *Neospora caninum*. In agreement with the *in silico* observations, sera of known *T. gondii* infected mice and goats recognized recombinant SAG2A, whereas no serological cross-reactivity was observed with samples from *N. caninum* animals. Additionally, the C-terminal end of the protein was able to down-modulate pro-inflammatory responses of activated macrophages and dendritic cells.

**Conclusions:**

Altogether, we demonstrate herein that recombinant SAG2A protein from *T. gondii* is immunologically relevant in the host-parasite interface and may be targeted in therapeutic and diagnostic procedures designed against the infection.

## Background

Toxoplasmosis is a zoonosis caused by *Toxoplasma gondii*, a coccidium member of the phylum Apicomplexa. *T. gondii* is present in a wide range of vertebrate hosts, including humans, which normally present asymptomatic infections. However, severe diseases may be observed in immunocompromised individuals and in congenital infection [1,2]. According to seroepidemiological data, approximately one third of the world population is chronically infected by the parasite, although prevalence may vary between 10% and to 80% depending on the economic, cultural and health status [3,4].

Several studies have been performed in order to understand the interactions between the parasite and its host cells [1,5-7]. Among the different classes of studied molecules, special attention has been spent on the glycosylphosphatidylinositol (GPI)-anchored proteins named SAG (surface antigens), SRS (SAG1-related sequences) and SUSA (SAG-unrelated surface antigens). The SRS family is divided into two major branches: the SAG1-like sequence family (SAG1, SAG3, SRS1-SRS4, BSR4) and the SAG2-like sequence family (SAG2ABCDXY) [1,8].

Genomic/Proteomic research within the *T. gondii* model has been very useful for the understanding of cell invasion mechanisms, cell cycle and immune evasion [9-12]. Protein modeling has been broadly used nowadays [13-15]. It is used to discover the spatial organization of a protein by prediction of molecular interactions, based on the crystal structure of relatively similar amino acid sequences, which may provide relevant data on its function and active sites. An example for the application of such techniques is the knowledge generated on the structural characterization of the Moving Junction (MJ), a complex structure produced by the parasite that is essential for host cell invasion [14,16].

In this study, we aimed to evaluate the interplay between the predicted three-dimensional structure of SAG2A protein and the immune system of its hosts, as shown here for mice and goats. Taken together, our results suggest that surface SAG2A protein contains an active C-terminal region that interacts directly with innate and adaptive immune mechanisms.

## Methods

### Ethics statement

Maintenance and care of mice were performed according to The Ethical Principles in Animal Research adopted by the Brazilian College of Animal Experimentation (COBEA) and was approved by the Ethical Commission of Ethics in Animal Research of the School of Medicine of Ribeirão Preto, University of São Paulo (CETEA-FMRP/USP), under protocol number 059/2007.

### Reagents

Reagents for cell culture were obtained from Life Technologies (Carlsbad, CA, USA) and Sigma-Aldrich (St. Louis, MO, USA); Reagents for detection of mouse IL-12p40 and TMB substrate were acquired from Becton and Dickinson (BD, San Diego, CA, USA). Lipopolyssacharide (LPS from *Salmonella typhymurium*), Griess reagent (sulfanimide and naphtylethylenediamine) and bicinchoninic acid (BCA) were acquired from Sigma-Aldrich (St. Louis, MO, USA). Recombinant cytokines GM-CSF, IL-4 and IFN-γ were acquired from Peprotech (Rocky Hill, NJ, USA). Monoclonal antibodies and irrelevant mouse IgG were purified using immobilized protein G-sepharose columns (Sigma). For protein concentration and ultrafiltration we used the Amicon system (Millipore, Billerica, MA, USA). Antigens were preserved from degradation by the addition of a protease inhibitor cocktail (Complete, Roche Applied Science, Mannheim, Germany). Recombinant proteins were determined as endotoxin free by a modified Limulus amebocyte lysate assay (LAL, BioWhittaker, Walkersville, MD, USA).

### Mice

Six- to eight-week old wild type (WT) C57BL/6 background mice were supplied by the Department of Biochemistry and Immunology, School of Medicine of Ribeirão Preto, USP, Ribeirão Preto, Brazil, and were bred and maintained at the institutions’ animal facilities, with food and water *ad libitum*.

### Cell culture, parasites and antigens

*T. gondii* (RH and Me49 strains) tachyzoites were maintained in HeLa cell lines (ATCC/CCL-2; American Type Culture Collection, Manassas, VA, USA) grown in RPMI 1640 medium supplemented with 2% fetal calf serum at 37°C in a 5% CO_2_ air environment. Parasites were harvested by scraping off the cell monolayer 5 days after infection and were purified by forcible extrusion through a 27-gauge needle and centrifugation (45 x *g*, 1 min, 4°C) to remove host cell debris.

### Construction of plasmid, expression and purification of recombinant proteins

*T. gondii* genomic DNA from RH strain tachyzoites was isolated as previously described [17], and the construction of plasmids, expression and purification of recombinant SAG2A (rSAG2A) and truncated protein at position 135 (rSAG2A^∆135^) were produced as described elsewhere [18]. Briefly, native SAG2A coding sequence was obtained in a public database (GenBank: AAO72427.1; [19]). Signal peptide and predicted GPI anchor were removed, and the template used for protein expression comprised of amino acids between 30 and 156 of the deposited sequence. For cloning and expression of the recombinant proteins, we used pET28a vector and *E. coli* Rosetta DE3 strain. To remove possible contamination from the process, protein purification was performed as previously described [20]. Briefly, Triton X-114 was added to purified SAG2A fraction to a final concentration of 1%. The mixture was incubated at 4°C for 30 min, with constant stirring to ensure a homogenous solution. The sample was then transferred to a 37°C water bath, incubated for 10 min and centrifuged (13,000 x *g*, 10 min, 25°C). The upper aqueous phase containing the protein was carefully removed and again subjected to Triton X-114 phase separation for at least two cycles. Additionally, the aqueous phase containing the protein was loaded into a Sephadex G75 column (1 × 50 cm) that had been previously equilibrated with 10 mM Tris–HCl pH 7.4 containing 0.5 M NaCl. The chromatography was performed with a flow rate of 3.5 ml/min and 500 μl fractions were collected and monitored at 280 nm OD. The fractions with high absorbance values were analyzed by SDS-PAGE, and the corresponding fractions were combined and concentrated by ultrafiltration. After purification, the protein concentration was determined by BCA method and screened for the presence of endotoxins by LAL assay.

### Protein modeling

To construct three-dimensional structures of SAG2A, modeling was based on structures solved by X-ray crystallography or RMN using the algorithm blastp and PSI-Blast (http://www.ncbi.nlm.nih.gov/BLAST) [21], using the matrix of alignment Blossum62 [22]. Sequences of the desired proteins were obtained at Protein database from the National Center for Biotechnology Information (NCBI, http://www.ncbi.nlm.nih.gov/protein) and at The Toxoplasma Genome Resource (ToxoDB, http://www.toxodb.org). The search for patterns was configured to use only the PDB database (http://www.pdb.org), which owns all three-dimensional structures of proteins from data of X-ray crystallography or NMR already published. For the construction of the models, we considered only those with identity above 25% and E-values of less then e.10^-5^. The verification of motif sequences belonging to the active site of molecules, such as protein family, was also taken into consideration in the choice of templates. The template chosen for the construction of three-dimensional models of *T. gondii* proteins SAG2A, as well as SAG2A orthologue from *N. caninum* (NcSAG2A; ToxoDB: NCLIV_035760), was the crystal structure of SAG1 (PDB: 1KZQ; [23]), which presents the conserved SAG superfamily motif and presented at least 35% identity with the modeled proteins. Construction of SAG2A three-dimensional model followed the sequence used for recombinant protein synthesis. The three-dimensional structure for BSR4 protein was analyzed according to its deposited crystal structure (PDB: 2JKS; [24]).

The initial structures obtained by homology modeling, were submitted to the validation process, using the programs PROCHECK 3.4 [25] and ANOLEA (Atomic Non-Local Environment Assessment) [26,27]. Validation by PROCHECK allowed the verification of stereochemical quality of the modeled structures, comparing them with other well refined structures at a resolution of 2.0 Å, as well as the residue to residue analysis of these structures. In this analysis, Ramachandran graphics [28] and other plots were generated for the validation of the three dimensional structure, plotting angles and torsion of chi1 and chi2 for all residues, planarity of peptide bonds, poor interaction between unconnected atoms, binding strength of hydrogen in the main chain and side chain properties [25]. Through validation by the ANOLEA, provided by Swiss-Model server (*swissmodel.expasy.org/anolea*), the total energy values of the models of the SAG2A proteins were calculated. This software evaluated the non-local environment of each heavy atom of these molecules. The interaction energy of each pair of atoms in their non-local environment was calculated using the potential strength dependent on the distance, within a radius of 7 Å for each amino acid, which was compared with a database of 147 non-redundant protein chains, with a sequence identity above 25% and obtained from X-ray crystallography with a resolution below 3 Å [27]. The GPI anchoring site of SAG2A was predicted using online tool PredGPI (http://gpcr.biocomp.unibo.it/predgpi).

### Bone marrow-derived macrophages and dendritic cells

Bone marrow-derived macrophages (BMMs) were generated from bone marrow stem cells of C57BL/6 background mice, as previously described [29]. Briefly, stem cells were cultured on 10 cm-diameter polystyrene plates for 7 days in RPMI 1640 medium containing HEPES 15 mM, 2 g of sodium bicarbonate/liter, and 1 mM L-glutamine and supplemented with 20% heat-inactivated fetal calf serum (FCS) and 30% L929 cell conditioned medium (LCCM). Differentiated BMMs were removed from the substrate by vigorous pipetting of ice-cold phosphate-buffered saline. Cells were counted and added (2 x 10^5^) to 96-well culture plates for the experiments.

Bone marrow-derived dendritic cells (BMDCs) were generated as previously described [30]. Briefly, bone marrow stem cells were cultured (7 x 10^5^ cells/well) in 24-well culture plates in RPMI medium supplemented with 10% CFS, 30 ng/ml of murine granulocyte–macrophage colony-stimulating factor (GM-CSF) and 10 ng/ml of murine recombinant IL-4. On days 3 and 6, supernatants were gently removed and replaced with the same volume of supplemented medium. On days 7–8, non-adherent cells were removed and plated on 96-well culture plates prior to stimulation.

### In vitro stimulation

In all *in vitro* experiments, cells were cultured in 96-well culture plates, in quadruplicate, using incomplete RPMI medium in the presence of *T. gondii* RH or Me49 strain tachyzoites, at different parasite:cell ratios (multiplicity of infection – MOI) and concentrations of recombinant SAG2A proteins, specific monoclonal antibodies (A4D12; [18]), or medium alone (negative control) at 37°C and 5% CO_2_ for 24 hours. BMMs pre-incubated with antigens or live parasites were stimulated with LPS and IFN-γ for further 48 hours, while BMDCs were stimulated with LPS for further 24 hours. After this period, the culture supernatants were removed for cytokine and nitric oxide measurements.

### Cytokine quantification by ELISA

The concentrations of IL-12p40 in cell culture supernatants were measured by commercial kits, according to the manufacturer’s instructions. Plates were read in a plate reader (Molecular Devices, Sunnyvale, CA, USA) at 450 nm. Cytokine concentrations were calculated from standard curves of murine recombinant cytokines. The detection limits for the assay was 15.6 pg/ml.

### Measurement of nitric oxide production

Nitric oxide production was measured by nitrite concentration in supernatants obtained from stimulated BMMs and BMDCs cultures by Griess assay [31]. Briefly, Griess reagent (sulfanimide 1% and naphtylethylenediamine 0.1%, 1:1) was added to the culture supernatants. Absorbance was measured at 540 nm. NO concentration was determined using standard curve established with a 200 μM sodium nitrite solution. The sensitivity limit was 1.563 μM.

### Immunoassays

ELISA and Western blotting protocols using recombinant SAG2A (rSAG2A) were performed for the detection of IgG antibodies present in serum samples of experimentally infected mice and naturally infected goats to *T. gondii* and *N. caninum*, as previously described [32,33]. Antibody titers were expressed as ELISA index (EI), according to the following formula: EI = OD sample/OD cut off, as described elsewhere [34]. Samples with EI values ≥ 1.2 were considered positive.

### Statistical analysis

Two-way ANOVA followed by Bonferroni posttests were applied to compare groups in relation to NO, IL-12p40 production. In all measurements, differences were considered significant when *p* < 0.05. All experiments were performed at least three times, and executed in different days. Statistical analysis of the data obtained was carried out using GraphPad Prism software (GraphPad, La Jolla, CA, USA).

## Results

**Figure 1:**
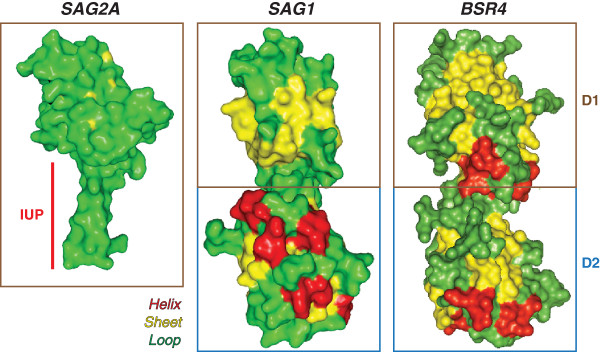
**Structural modeling of surface SAG2A protein and comparison with related *Toxoplasma gondii *antigens SAG1 and BSR4.** Structural modeling of surface SAG2A protein and comparison with related Toxoplasma gondii antigens SAG1 and BSR4. Three-dimensional models of SAG-related surface proteins. The predicted model of the single domain of SAG2A, with its unfolded C-terminal end (red highlight). This feature is not observed in the structure of the external (D1) and internal domains (D2) of the related tachyzoite surface proteins SAG1, and in correlated bradyzoite antigen BSR4.

After performing the *in silico* analysis of the three-dimensional model of SAG2A and known crystal structures (SAG1 and BSR4), we observed that the single domain of SAG2A protein presents a predicted disordered amino acid sequence forming a prominent loop in its C-terminal end (Figure 1). Interestingly, this same conformation was not observed in the crystal structure of tachyzoite-related surface proteins SAG1 [23] and in bradyzoite-related BSR4 [24]. Both proteins present a more complex structure - composed of two distinct domains (external and internal domains; D1 and D2, respectively) – and are disposed as dumbbell shaped monomers. The unique domain of SAG2A presented higher similarity to D1 of the other SAG-related proteins (Figure 1).

**Figure 2:**
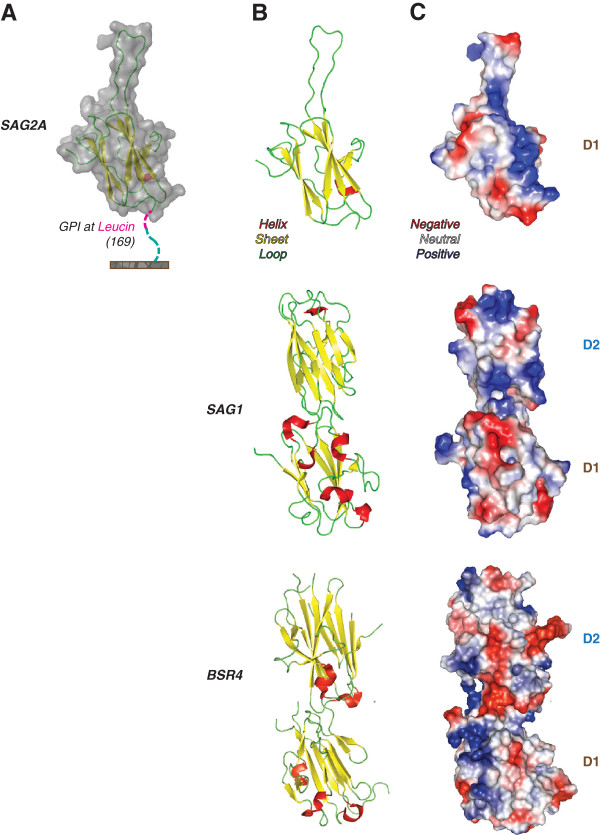
**Anchorage site, carbon structure and charge distribution of SAG2A protein.** (**A**) SAG2A anchorage in *Toxoplasma gondii*’s surface surface by glycosyl-phosphatidylinositol (GPI) was predicted to be in a leucin at position 169 of its amino acid sequence, located at the C-terminal end of SAG2A. (**B**) The modeled carbon structure of SAG2A evidences a disordered amino acid sequence, absent in the SAG1 and BSR4 proteins. (**C**) The C-terminal end of SAG2A presents a relevant hydrophobic portion, with distinct polar amino acids at positions 134 and 137.

Anchorage by glycosylphosphatidylinositol (GPI) in SAG2A was predicted in a leucin at position 169 of its amino acid sequence, within C-terminal region of SAG2A (Figure  2A), in contrast with the anchorage of SAG1 and BSR4 proteins, which occurs in D2 domains [23]. The monomer is maintained by eight beta-sheets producing a structure similar to the internal domains of the other SRS members analyzed, although SAG2A presents a unique extended loop (Figure 2B). Concerning the polarity of SAG2A molecule, it could be observed that its three-dimensional structure did not present a predominance of charges in its surface. However, the loop region within the C-terminal end displays a high hydrophobic portion with only two terminal polar amino acids, a positive and a negative residue (Figure 2C).

**Figure 3:**
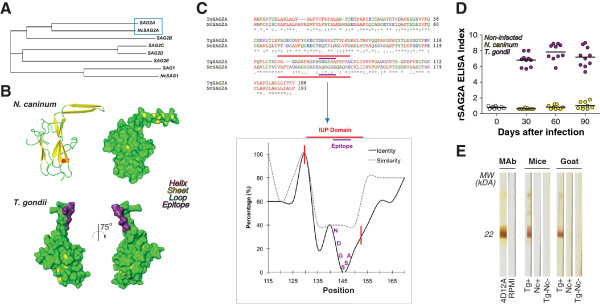
**Orthologue SAG2A from *Neospora caninum *presents distinct conformation of the C-terminal end and does not share immunodominant B cell-epitope with *T. gondii.*** (**A**) The consensus tree of SAG2 and SAG1 protein sequences demonstrates higher proximity of SAG2A with its predicted orthologue from *Neospora caninum* (NcSAG2A). (**B**) Modeling of NcSAG2A suggests that overall structure of the orthologues is similar, although its loop is composed with beta-sheets instead of the largely disordered structure of *T. gondii’s* protein. Additionally, the B cell-epitope sequence found in SAG2A (purple highlight) is not present in its orthologue. (**C**) Sequence alignments produced by ClustalW with SAG2A orthologues demonstrate the percentage of identity and similarity between these proteins, found to be low in the loop sequence and practically nonexistent in the B cell-epitope region of the amino acid sequence. The unfolded C-terminal end of *T. gondii* SAG2A is highlighted in blue, which includes an immunodominant epitope NDGSSA highlighted in red. (**D**) Reactivity of IgG antibodies from naïve mice and mice experimentally infected with *N. caninum* or *T. gondii* against recombinant SAG2A protein of *T. gondii*. Sera reactivity was expressed as ELISA index (EI). (**E**) Recognition profile of recombinant SAG2A by serum samples from experimentally infected mice and naturally infected goats with *T. gondii* and *N. caninum* in Western Blot.

Phylogenetic analysis of the SAG2A sequence revealed its resemblance to other members of the SAG2 cluster from *T. gondii*, although it presented higher identity with predicted orthologues from *N. caninum* (NcSAG2A), a closely related coccidian parasite (Figure  3A). Modeling of NcSAG2A suggested similar overall structure of the orthologues, although the models predicted for both proteins differed in the structural organization of the loop present at the C-terminal end of the molecules (Figure 3B). While SAG2A from *T. gondii* presents a largely disordered loop, NcSAG2A presents a loop sequence composed with beta-sheets.

SAG2A is a serologically immunodominant protein, mainly recognized in the acute phase of the infection by *T. gondii*[32]. Noteworthy, we have previously characterized a B cell epitope (NDGSSA) in this protein using monoclonal antibodies (A4D12 mAb; [18]). In the present work, our results suggest that this immunodominant epitope is located within the C-terminal end of the amino acid sequence (purple highlight, Figure 3B). Overall, sequence alignment of the SAG2A orthologues resulted in a high percentage of identity and similarity between these proteins, although NcSAG2A differed significantly from *T. gondii* SAG2A in the described IUP domain (Figure 3C). In order to check if the lack of homology in the loop sequences of SAG2A orthologues could confer unique antibody responses, we assayed serum samples of infected animals from both parasites against recombinant *T. gondii* SAG2A protein (rSAG2A). IgG antibodies from mice reacted strongly with rSAG2A by ELISA, after 30, 60 and 90 days of experimental infection with *T. gondii*, while no reaction was observed in samples from mice infected with *N. caninum* (Figure 3D). The same serological specificity was observed by Western blotting analysis of samples obtained from naturally infected goats – a ruminant known to be clinically affected by both parasites [35]. As expected, only samples from animals infected with *T. gondii* recognized the 22 kDa rSAG2A (Figure 3E).

**Figure 4:**
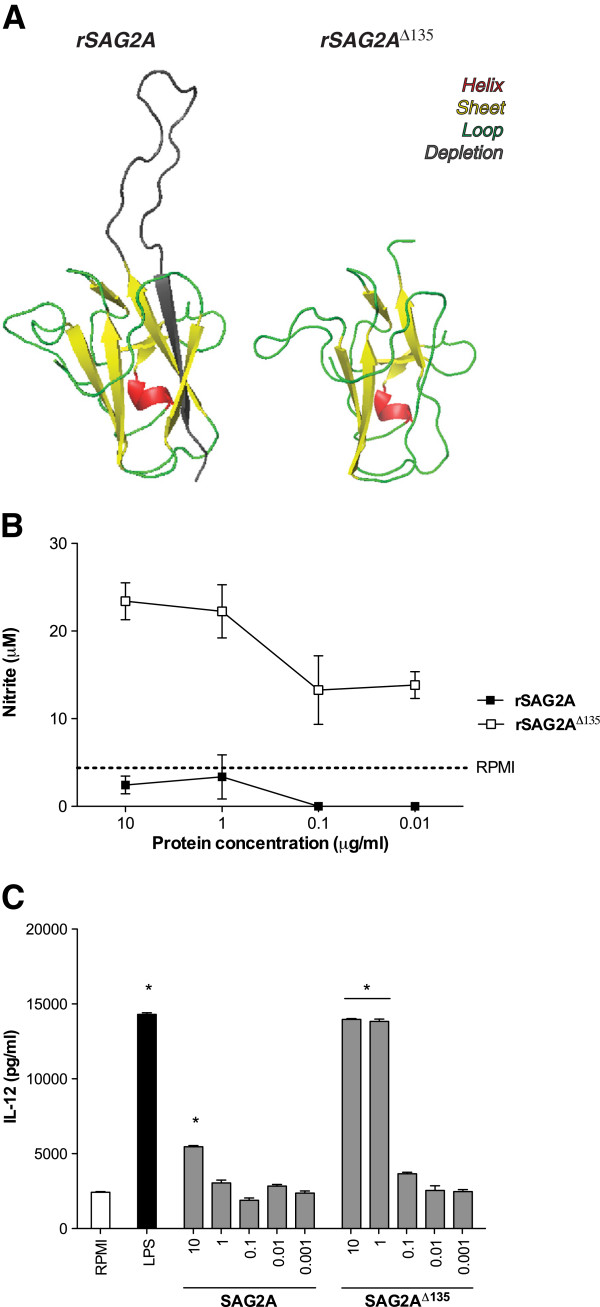
**C-terminal end of SAG2A protein interacts with innate immune response.** (**A**) Three-dimensional model of recombinant SAG2A (rSAG2A) and the truncated form of the protein (rSAG2A^∆135^). Structural analysis shows that depletion of the C-terminal end did not affect the overall predicted three-dimensional structure or distribution of positive and negative charges along the protein. (**B**) Bone marrow-derived macrophages (BMMs) and (**C**) dendritic cells (BMDCs) were treated with different concentrations of rSAG2A and rSAG2A^∆135^ for 48 and 24 h, prior to determination of nitrite (**B**) and IL-12p40 levels (**C**), respectively. As controls, cells were left untreated (RPMI) or exposed to LPS (1 μg/ml) in similar time spam. Results are presented as mean ±SEM. Dashed lines indicate mean values obtained for untreated BMMs. * Statistical significance (p < 0.05) in relation to untreated controls.

*T. gondii* is known to actively interfere in macrophage functions, through the blockage of innumerous LPS-inducible cytokines, modulation of the host gene expression, inhibition of IL-12p40 and TNF-α, and suppression of nitric oxide (NO) production [36-38]. In that sense, we assessed whether SAG2A presented noted biological activities in innate immune cells. For that, we measured NO and IL-12 production in bone marrow derived macrophages (BMM) and dendritic cells (BMDC), exposed to rSAG2A, as well as a truncated protein lacking its C-terminal end (rSAG2A^∆135^, Figure 4A). Treatment of cells with rSAG2A or rSAG2A^∆135^ induced very distinct patterns of response in innate cells. While the intact protein induced almost undetectable levels of NO and IL-12p40, rSAG2A^∆135^ triggered proinflammatory responses in BMMs (Figure 4B) and BMDCs (Figure 4C).

**Figure 5:**
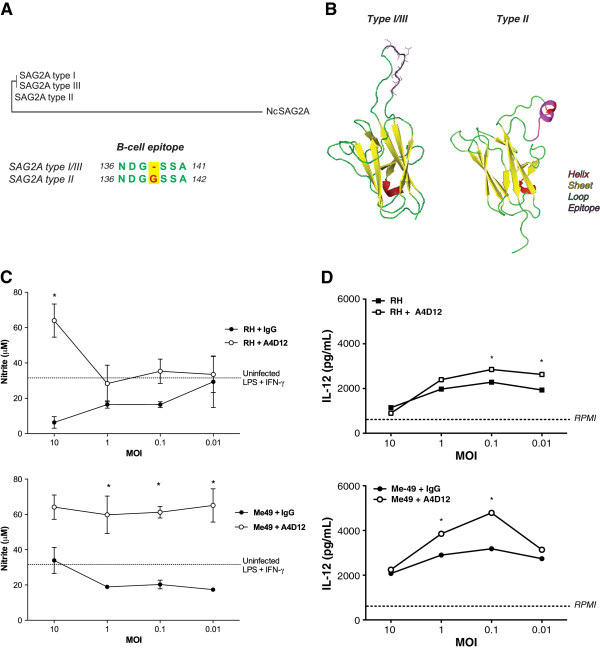
**Strain-dependent SAG2A polymorphisms in the C-terminal end alter protein conformation and regulatory features.** (**A**) The consensus tree of SAG2A from the three clonal strains (I, II, III) of *Toxoplasma gondii* and the orthologue expressed in *Neospora caninum*. Alignment of the immunodominant epitope region shows that type II SAG2A displays a single additional glycine (position 142) within its IUP domain sequence*.* (**B**) Modeling of SAG2A from type I/III strains with the epitope region highlighted within the C-terminal end. The addition of glycine is responsible for significant changes in the SAG2A structure in type II strains, creating a predicted coil. (**C**) Bone marrow-derived macrophages (BMMs) and (**D**) Bone marrow-derived dendritic cells (BMDCs) were previously exposed to different parasite:cell ratios (multiplicity of infection – MOI) with RH and Me49 strain tachyzoites, in the presence of A4D12 monoclonal antibody or irrelevant IgG for 24 h. BMMs activation were promoted by LPS (10 ng/ml) + IFN-γ (100 ng/ml), 48 h prior to determination of nitrite levels. BMDC activation was promoted by LPS (1 μg/ml), 24 h prior to determination of IL-12p40 levels. Results are presented as mean ±SEM. Dashed lines indicate mean values for experimental controls following description. * Statistical significance (p < 0.05) related to antibody treatments.

It is well known that *T. gondii* possesses great strain variability, usually represented by the expression of distinct isoforms of membrane-bound and secreted proteins [8,18,39]. That altered expression may correspond to differences in virulence factors to human and murine hosts [40,41]. Phylogenetic analysis of the protein sequence, using ToxoDB database, revealed that SAG2A protein sequences expressed in the major clonal *T. gondii* strains (I, II and III) present high identity. However, as previously described [40,42], type II strains display a single additional glycine at position 139, if compared with type I/III strains (Figure 5A). Observation of the predicted three-dimensional models suggests that the additional glycine in SAG2A from type II strains promotes significant changes in protein structure, creating a predicted coil along the C-terminal end of the sequence (Figures 5B). That structural modification is located in the predicted B-cell epitope, but it does not alter its antigenic recognition by directed monoclonal antibody, as described previously [18].

In order to check whether these predicted structural modifications within the IUP domain would alter the protein ability to modulate innate cell activation, we opsonized SAG2A IUP domains in tachyzoites of RH and Me49 strains with A4D12 mAb. As shown in Figure 5C, pretreatment of RH tachyzoites with A4D12 mAb resulted in increased production of NO by infected BMMs stimulated with LPS and IFN-γ. However, mAb sharply reverted Me49-promoted downregulation of NO, while that effect was not so evident in treated RH tachyzoites. The same pattern of events was observed for IL-12p40 production by BMDCs, where Me49 parasites blocked with A4D12 mAb were able to induce greater production of the cytokine, if compared to RH treated tachyzoites (Figure 5D). These observations indicate that the modulatory potential of SAG2A expressed in both parasite strains is distinct, which may be related to the differences displayed within its C-terminal domains.

## Discussion

*T. gondii* surface antigens are crucial in the initial interactions with host cells, mediating events such as attachment and invasion [43,44]. Additionally, the ability of these proteins to induce cellular and serological immune responses has been in discussion since the early 1990’s [45,46].

Our results indicate, through structural modeling prediction, that SAG2A presents unique features in the C-terminal end of its amino acid sequence, which resembles the features of Intrinsically Unstructured Proteins (IUP). IUPs have been identified as probable causative elements of neurodegenerative diseases and viral virulence factors, due to their ability to interact with different molecules within the cells [47,48]. Previous studies have shown that some eukaryotic genomes present over 20% of residues with similar characteristics [49]. IUPs differ from structured proteins especially in their functions, usually related to catalysis, membrane transport and reversible binding of small molecules, signal transduction, cell-cycle regulation, gene expression and chaperone action [50-52]. The identification of proteins with disordered regions, and the mapping of their exact location within those molecules, can be an important step in the selection of antigenic targets due to their importance in microbial metabolism [49]. Genomic predictions have estimated that Apicomplexan parasites present IUP domains in a higher frequency than bacteria and archaea, and *T. gondii* is one of the parasites with most occurrences, alongside *P. falciparum*, *P. vivax* and *P. knowlesi*[53].

Our present work locates a previously predicted B cell linear epitope [18] within this predicted IUP domain of SAG2A, which corroborates with our previous studies on immunodominance of the protein and its potential as an acute phase marker for the diagnosis of human toxoplasmosis [32,33,54]. In addition, we show here that recombinant SAG2A was able to specifically detect *T. gondii*-induced antibodies in experimental and natural infections eliminating potential serological cross-reactivity with closely related *N. caninum*, a long-lived setback within the veterinary field [55-57]. In a similar manner, other authors have confirmed that SAG2A protein can be used for diagnostic procedures, and also include this protein as a target in the development of subunit vaccines against *T. gondii* infection [58]. However, strategies involving SAG2A as an immunogen yield divergent results, and some failed to protect susceptible mice from lethal infections [59-61]. Based on our results, we believe that the inability to establish SAG2A as an immunogen in those studies may be due to the modulatory properties of its C-terminal end and we suggest that r*SAG2A*^*∆135*^ should be tested in those experimental models.

Down regulation of innate immune response induced by parasite antigens, endogenous ligands or other microbial molecules has been extensively assessed through the last decades [62,63], although a very small number of parasitic proteins and their mechanisms of action have been identified with those properties. The results of this study suggest that the unusual C-terminal end predicted in SAG2A may directly affect the activation status of macrophages and dendritic cells. The absence of this C-terminal end in SAG2A, observed through antibody inhibition and depleted recombinant proteins, reverted the parasite-induced suppression of nitric oxide and IL-12p40 production by LPS - an experimental model based on studies that demonstrate the potent suppression of LPS-induced activation by *T. gondii* in cells of the immune system [35].

Parasite strategies to fine-tune the immune response of the host may be explained by its necessity to maintain the host alive during the acute phase of infection, in order to propagate and convert to latent stages inside muscular and nervous tissues, waiting for the infected host to be preyed upon by a carnivorous feline definitive host [39,64,65]. In this sense, with an interest in understanding the elaboration of the immune response through interaction between host and parasite, significant efforts have been placed on identifying parasite-derived ligands that may activate or deactivate immune responses triggered by the infection process [5,6,16,66]. Activation of innate immunity by *T. gondii* is mainly triggered by the Toll-like and chemokine receptor agonists [1,67]. Recent studies have demonstrated that TLR11/TLR12 activation via parasite actin-related profilin induces high production of IL-12p40 and IFN-γ, key cytokines required for host protection during toxoplasmosis [68,69], although innate pathogen sensing through this activation pathway is not preserved in all animal species [70].

Despite of its evolutionary need for host survival and maintenance of its epidemiological chain, *T. gondii* possesses a great variety of mechanisms to suppress pro-inflammatory responses, in order to evade the microbicidal mechanisms induced by molecules, such as NO, GTPase and inflammatory cytokines, which can be highly effective in parasite clearance [37,71,72]. A set of polymorphic kinases from *T. gondii* have recently been identified as virulence factors - namely ROP5, ROP16 and ROP18 - secreted from its rhoptries into the host cell cytoplasm [21,73]. Much attention has been given to ROP16, since it activates directly STAT3 and STAT6 in the host cell cytoplasm, inhibiting the production of proinflammatory mediators [64,73,74]. The control of proinflammatory cytokine production is in great part promoted by the IL-10/STAT3 signaling cascade during *T. gondii* infection [62]. As one might expect, the activation of this signaling pathway minimizes host cell damage, however, it prevents effective parasite clearance. Although the rhoptry kinases present great modulatory potential, it is not possible to affirm that these proteins are solely responsible for the evasion mechanisms displayed by the parasite, since some contradictions have been reported in different models of analysis [64].

We have also observed that antibody inhibition of SAG2A in *T. gondii* strains induced differential immune response profiles in innate cells. Genetic distinctions found between *T. gondii* strains have been implicated in the pathogenesis of the disease, through host immune cell modulation [41,75]. Disparity between *T. gondii* strains commonly occurs due to amino acid polymorphisms, rather than quantitative differences. It has been shown that ROP16 presents a single amino acid substitution in the kinase domain between type I and type II strains, which is sufficient for differential STAT3 activation [76]. Another example described recently are the polymorphisms within strains in the GRA15 locus that interferes directly with NF-κB activation in the host cell, leading to differential immunopathology in the mouse model [77,78]. Understanding the role of each of these proteins may be an important step to unravel the host-parasite interactions during toxoplasmosis. Polymorphisms at the SAG2A locus are found to be between 1% and 5%, and antibodies against the protein isolated after natural infections were shown to be strain-specific, even though all three strain types (I, II, III) express approximately equal levels of the SAG2A protein, suggesting that although polymorphic, epitopes within SAG2A are still highly immunogenic and immunodominant [40].

## Conclusions

In conclusion, we found that the C-terminal end of *T. gondii* surface protein SAG2A interacts with distinct immune mechanisms, sheltering a known B cell epitope, also being potentially involved with immune evasion of macrophages and dendritic cells. In this context, further studies are needed in order to confirm the exact intracellular mechanisms activated by its C-terminal end. The confirmation of these results could provide relevant information for the development of new prophylactic and therapeutic approaches toward toxoplasmosis.

## Competing interests

There are no potential competing interests related to the data herein presented.

## Authors’ contributions

Conceived and designed the experiments: AGMJr JPCJr CPP DAOS JRM TWPM. Performed the experiments: AGMJr THSC MVS FMS DAOS TWPM. Analyzed the data: AGMJr JPCJr THSC FMS CPP DAOS JRM TWPM. Wrote the paper: AGMJr JPCJr TWPM. All authors read and approved the final version of the manuscript.
